# Pathways Linking ICT Use to Chronic Disease Self-Management Among Older Adults with Comorbidities in Shanghai, China

**DOI:** 10.3390/healthcare13131626

**Published:** 2025-07-07

**Authors:** Qingru Chen, Ke Gong, Zhijun Bao, Yuanfang Yin, Lirong Zhao, Yan-Yan Chen

**Affiliations:** 1 Medical Social Work Department, Huadong Hospital, Fudan University, Shanghai 200031, China; qingruchen05@163.com (Q.C.); hdsgb2013@163.com (K.G.); 2 Shanghai Institute of Geriatrics and Gerontology, Shanghai 200031, China; zhijunbao@fudan.edu.cn; 3 Department of Geriatrics, Huadong Hospital, Fudan University, Shanghai 200031, China; 4 Huadong Hospital, Fudan University, Shanghai 200031, China; yinyuanfang110@126.com; 5 Department of Nursing, Huadong Hospital, Fudan University, Shanghai 200031, China; huadongzlr@163.com; 6 Department of Social Work, Fudan University, Shanghai 200437, China

**Keywords:** chronic disease, health literacy, information and communication technology use, social support, self-management behavior, older adults, mediation effect

## Abstract

**Background:** The mechanisms through which information and communication technology (ICT) use influences chronic disease self-management remain unclear. **Method:** This cross-sectional investigation examined the mediating effects of health literacy, social support, and self-efficacy on the association between ICT use and self-management behaviors among older adults (≥60 years) with comorbidities in China (*n* = 520). The participants were recruited from a tertiary hospital in Shanghai (July 2023–June 2024), and data on sociodemographics, self-management, health literacy, social support, self-efficacy, and ICT use were collected via structured questionnaires. **Results:** Pearson’s correlation analysis revealed a significant association between ICT use, health literacy, social support, self-efficacy, and chronic disease self-management (*p* < 0.001). Multiple mediation modeling revealed a direct positive effect of ICT use on self-management (*b* = 1.3314, 95% CI = 0.6629, 2.0002). Furthermore, significant indirect effects were observed, mediated independently by both health literacy and social support. Additional serial mediation pathways included health literacy → social support, health literacy → self-efficacy, social support → self-efficacy, and a comprehensive pathway from health literacy through social support to self-efficacy. **Conclusions:** These findings collectively indicate that ICT use directly enhances chronic disease self-management among older adults with comorbidities. Moreover, ICT use indirectly improves self-management by enhancing health literacy, augmenting social support, and bolstering self-efficacy.

## 1. Introduction

Information and communication technology (ICT) has been used to support chronic disease self-management and patients’ empowerment, primarily through the Internet [1]. ICT interventions in healthcare have demonstrated cost-effectiveness in monitoring and controlling various chronic diseases and issues, including congestive heart failure, stroke, chronic obstructive pulmonary disease, diabetes, hypertension, asthma, cancer, depression, and chronic pain [2,3,4,5]. The use of ICT for self-management has become a focal point of domestic and international research [6,7], making it a crucial perspective in research related to self-management. Kang and colleagues [8] demonstrated that combining ICT and health coaching improved self-management, increased exercise, and promoted several healthy behaviors. The organizers of the Health Social Partnership Program in Hong Kong successfully employed ICT-based self-management support to improve patients’ confidence and behavioral capacity through personalized health education and support delivered via video conferencing and telephone [9]. Compared to the Hong Kong model, the adoption and refinement of health ICT in mainland China have not yet reached a high level. Subsequent services require more empirical evidence.

Comorbidities, the co-occurrence of two or more chronic diseases, present significant management and treatment challenges due to potential disease interactions [10,11]. Affecting approximately 50% of individuals aged 65 or older globally [12], their prevalence increases sharply with age. The necessity of managing multiple conditions can render therapeutic strategies repetitive, inefficient, burdensome, and unsafe due to coordination deficits [13]. Multimorbidity significantly diminishes quality of life [14], reduces life expectancy, elevates disability and mortality risks [15], affects mental health, increases the use and costs of healthcare resources, and imposes economic strain [16]. Given their compromised physical functioning and the protracted nature of managing complex conditions after hospitalization, older adults with multimorbidity face heightened demands for self-management [17].

Effective comorbidity self-management requires broader informational and skill acquisition than the management of a single chronic condition, with the internet serving as a common information source. However, the effectiveness of ICT use for self-management in this population remains contested. Although studies such as those conducted by Kawai et al. [18] and Waki et al. [19] have reported significant benefits of ICT-enhanced systems for self-management among individuals with obesity and diabetes, Kang et al. [9] found no significant difference in self-management behavior when ICTs were employed as a standalone intervention.

Studies have pointed out the importance of ICT use in promoting self-management and improving quality of life among older adults with chronic disease. Older adults’ psychosocial characteristics, such as greater health literacy, more social support, and self-efficacy, have been considered essential factors significantly associated with greater self-management behavior [20].

Health literacy is crucial for fostering self-management behaviors for chronic diseases [21,22], as it is a significant determinant of health behavior and outcomes [23]. Defined by Nutbeam [24] as “cognitive and social skills related to the ability of individuals to access, understand, and use health information and to develop and maintain health status,” its importance in self-management is well-established.

Although strong health literacy is valuable for making independent health decisions, many individuals, particularly older adults or those with multiple diseases, often rely on social support networks (e.g., family members, friends, neighbors, relatives, and others) for effective health management [25], especially when healthcare needs arise. This access to social support demonstrably enhances mental well-being [26] and improves treatment adherence [27], which are crucial for successful self-management. Notably, these networks’ association with the self-management of chronic kidney disease is even stronger than that for health literacy [28]. This underscores the significant role that social support plays in enabling effective self-management, especially for those facing age-related challenges or the complexities of multiple chronic illnesses.

Self-efficacy, an individual’s belief in their capacity to successfully execute a specific activity [29], plays a key role in effective self-management. It is a core belief that drives motivation for change, the ability to overcome obstacles, and the sustainability of new behaviors [30]. Studies have consistently shown that it has a positive correlation with chronic disease self-management [31,32]. Furthermore, evidence suggests that social support indirectly influences self-management behaviors by enhancing overall self-efficacy [33]. Interventions that strategically incorporate self-efficacy enhancement have effectively improved these behaviors [34].

Previous studies have found positive associations between self-management and ICT use, health literacy, social support, and self-efficacy, individually and in various combinations. Yet, the underlying mechanism governing the relationship between ICT use and self-management remains unclear, particularly in terms of the functions mediated by health literacy, social support, and self-efficacy in older populations with comorbidities. Specifically, research examining these relationships among older adults in developing countries like China has not yet been reported. Further investigation into their interactive effects on ICT-enabled self-management is needed. Therefore, examining the magnitude and significance of these pathways within a multiple mediation framework is crucial. In the framework of triadic reciprocal determinism, Bandura [35] clarified the dynamic interaction among individuals, environment, and behavior. Within this model, behavior emerges from a continuous interplay that evolves in response to changing social circumstances. It is reciprocally affected by the interplay of environmental factors (e.g., social support), personal factors (e.g., health literacy and self-efficacy), and behavioral factors (e.g., ICT use and self-management). Based on this theoretical foundation and the existing literature, we developed and tested a model that considered health literacy, social support, and self-efficacy as three mediators enhancing the effects of ICT use on self-management behavior among older adults with comorbidities. To the best of the authors’ knowledge, this study is the first to examine these variables together empirically. Based on the literature above, we proposed the following hypothesized pathways to describe the link between ICT use and self-management: a direct effect of ICT use on self-management and seven indirect (mediation) effects via health literacy; social support; self-efficacy; health literacy and social support; health literacy and self-efficacy; social support and self-efficacy; and health literacy, social support, and self-efficacy. In the subsequent section of this paper, we will examine the relationships between these variables based on questionnaire data collected from a tertiary hospital in Shanghai, China, and then further discuss our findings.

## 2. Methods

### 2.1. Study Design and Participants

We adopted a cluster-sampling strategy for this single cross-sectional survey. Data were collected from a tertiary hospital in urban Shanghai from July 2023 to June 2024. This hospital specializes in geriatric medicine, covering over 40 departments, including cardiology, respiratory medicine, gastroenterology, endocrinology, nephrology, and neurology, each with approximately 40 inpatient beds. Participants were mainly recruited from the endocrinology, nephrology, and neurology inpatient wards. The inclusion criteria were (a) being 60 or older; (b) having at least two chronic diseases; (c) being able to understand the questionnaire; and (d) having volunteered to participate. Patients with mental disorders, communication impairment, or serious illnesses and complications were excluded. The study team informed the patients of the aim, content, investigation procedures, and possibility to withdrawal from the study at any time. The paper-based questionnaires were administered by trained interviewers through face-to-face interviews, each lasting approximately 30 min and being voluntary. A comprehensive quality check of questionnaire completion was conducted prior to the participants’ dismissal, yielding very few missing values. Of the 538 older patients invited, 13 declined to participate, and 5 dropped out during the survey due to reasons such as ongoing treatment requiring too much time or unstable emotional states. Therefore, 520 valid questionnaires were returned, with a 96.65% response rate. This proportion is much higher than 70%, which has been suggested to be a robust response rate substantially reducing the risk of significant nonresponse bias in many fields [36]. This study was reviewed and approved by the Institutional Review Board of the research ethics committee of the hospital from which the participants were recruited (No. 2022K235).

### 2.2. Measures

#### 2.2.1. Health Literacy

The Health Literacy Scale-European Union-Q16 [37,38] was adapted to measure health literacy. It has 16 items in three subdomains: health care, disease prevention, and health promotion. Each item indicates perceived difficulty using a 4-point Likert scale (1 = very difficult to 4 = very easy). Higher scores suggest better health literacy. Cronbach’s alpha was set at 0.89.

#### 2.2.2. Social Support

The Brief Chronic Illness Resource Survey [39] was adapted to measure social support. Ten items were selected to measure sources of support for patients with chronic diseases, including family and friends, neighborhoods or communities, and organizations. This tool features a 5-point Likert scale (1 = not at all to 5 = a great deal). Higher scores indicate higher levels of social support. Cronbach’s alpha was set to 0.78.

#### 2.2.3. Self-Efficacy

The Self-Efficacy to Manage Chronic Disease measure was used to assess how confident each individual felt in undertaking self-management tasks regarding chronic diseases [40]. It has six items that assess confidence in managing fatigue, physical discomfort or pain, emotional distress, other symptoms or health issues, tasks or activities carried out to manage health conditions, and tasks other than using medication. Responses are given on a 10-point scale (1 = not confident at all to 10 = totally confident). Scale scores are standardized into a mean between 1 and 10, with higher scores representing greater confidence in chronic disease self-management. The measure has been translated into Chinese [41], and Cronbach’s alpha was set to 0.88.

#### 2.2.4. ICT Use

The European Survey on e-Health Use was adapted to measure ICT use [42]. The original questionnaire consisted of 88 items modified to suit the Chinese context. The final version assessed the number of ICT devices used, the number of health-related software products used, the purpose of using ICT and the frequency with which it is used, and the evaluation of ICT benefits. Cronbach’s alpha coefficient was set to 0.846. The final value used in the path analysis was calculated using principal component analysis.

#### 2.2.5. Chronic Disease Self-Management

In this study, the Partners in Health Scale was adapted to measure chronic disease self-management, covering disease knowledge, understanding of treatment options, involvement in disease management, appointment scheduling, medication adherence, health monitoring, and development of healthy habits [43]. A 9-point Likert scale was used, with higher scores indicating a higher level of self-management. Cronbach’s alpha coefficient was set at 0.915.

#### 2.2.6. Covariables

Some factors can produce spurious associations. Therefore, this study statistically accounts for demographic data, including age, gender, education, marital status, income level, living status, number of chronic diseases, activities of daily living, depression, self-rated health, and cognitive status.

### 2.3. Statistical Analyses

All analyses in this study were conducted using SPSS 23 for Windows (IBM Corporation, Armonk, NY, USA). For continuous variables, we used means, standard deviations, and medians with ranges, whereas categorical data were assessed using counts and percentages. The statistical significance level for all tests was set at *p* < 0.05.

**Figure 1:**
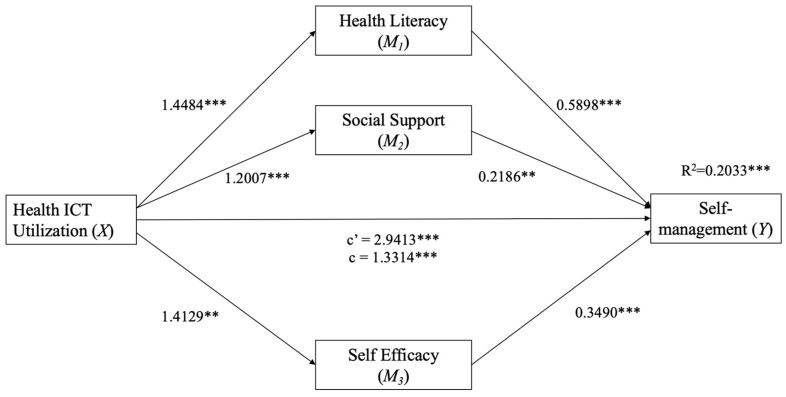
Path diagram illustrating the parallel mediation model. Each of the hypothesized mediators independently mediated the association between ICT use and self-management. Notes: c′ = direct effect of health ICT use on self-management; c = total effect of health ICT use on self-management. ** *p* < 0.01. *** *p* < 0.001.

**Figure 2:**
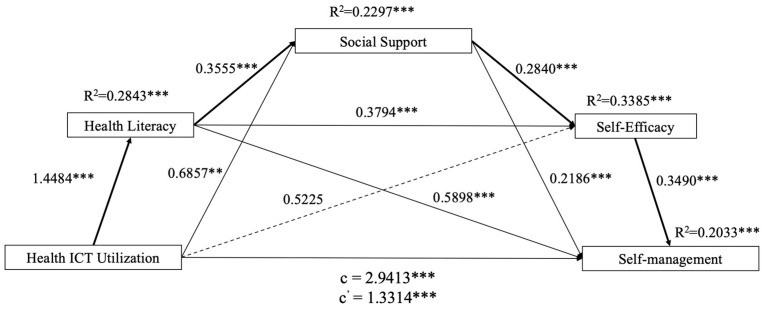
Path diagram illustrating the serial multiple mediation model with path coefficients. Multiple mediation modeling revealed a direct positive effect of ICT use on self-management. Significant indirect effects were observed, mediated independently by both health literacy and social support. Additional serial mediation pathways included health literacy leading to social support, health literacy influencing self-efficacy, social support affecting self-efficacy, and a comprehensive pathway from health literacy through social support to self-efficacy. Notes: c = total effect of ICT use on self-management, c′ = direct effect of ICT use on self-management. ** *p* < 0.01. *** *p* < 0.001.

For multiple mediation analysis, parallel and serial multiple mediation models were applied. First, a parallel multiple mediation model [44] (SPSS PROCESS macro version 3.5.3, Model 4) was employed with three mediators (health literacy, social support, and self-efficacy) to examine the indirect influence of ICT use on self-management (Figure 1). Recognizing that mediators may exhibit mutual influences and interactive effects, supplemental analysis using Hayes’ [44] serial multiple mediation model (SPSS PROCESS macro version 3.5.3, Model 6) was conducted to test the direct and indirect effects of the three interconnected mediators on the relationships between dependent and independent variables, following the hypothesized chains (Figure 2). The hypothesized mediation models were analyzed using the PROCESS macro with 10,000 bootstrap bias-corrected 95% confidence intervals (CIs). If the 95% bootstrap CIs excluded zero, indirect effects were considered statistically significant [44]. Following Hayes’ [44] recommendation, unstandardized regression path coefficients are presented to increase interpretability.

The following criteria were used to examine model fit: chi-square/*df* ≤ 2, *p* > 0.05, comparative fit index ≥ 0.95, and root mean square error approximation < 0.06.

## 3. Results

### 3.1. Participant Characteristics and Study Variables

The mean age of the participants was 70.8 years (*SD* = 6.3). The sample was 50.4% male, 79.6% married individuals, 58.1% high school graduates or individuals with higher qualifications, 89.0% individuals who lived with others, and 94.6% people earning more than RMB 2590 monthly. The mean number of chronic diseases was 3.71 (*SD* = 1.54), and the mean index of activities of daily living was 80.77 (*SD* = 15.32). The average depression symptoms score was 3.31 (*SD* = 3.84), and the average self-rated health score was 3.17 (*SD* = 0.83). Table 1 lists the mean scores for ICT use, health literacy, social support, self-efficacy, and self-management.

### 3.2. Correlations Among Study Variables

Table 2 presents the Pearson correlation coefficients among the study variables, revealing significant positive intercorrelations for all pairwise relationships.

### 3.3. Parallel Mediation Model

The findings presented in Table 3 highlight the parallel mediating effects of health literacy, social support, and self-efficacy on the relationship between ICT use and self-management behaviors among older adults with comorbidities. Specifically, the results indicate that each of the hypothesized mediators—health literacy, social support, and self-efficacy—independently mediate the association between ICT use and self-management.

In the parallel mediation model (Figure 1), employing bootstrapping with 5000 samples, the path coefficients (Figure 1 and Table 4) revealed a significant direct effect of ICT use on self-management (*b* = 1.3314, *SE* = 0.3404, *t* [520] = 3.9114, *p* < 0.001) and a significant total effect of ICT use on self-management when considering the three mediators concurrently (*b* = 2.9413, *SE* = 0.3851, *t* [520] = 7.6387, *p* < 0.001). The path coefficients presented in Table 4 show a significant overall indirect effect (*b* = 1.6099, 95% CI = 1.1188, 2.1314). Furthermore, each of the three mediators in the parallel mediation model exhibit a statistically significant contribution to the observed indirect effect (see Table 4 for path coefficients and CIs). Additionally, regression analysis (*R*^2^ = 0.4517, *F* [14, 505] = 29.7125, *p* < 0.001) indicated that the independent variable and three mediators collectively account for 70.92% of the variance in self-management.

### 3.4. Serial Mediation Model

The results obtained using the serial mediation model are shown in Figure 2. First, the hypothesized model’s fit to the data was assessed using AMOS, with evaluation based on the following criteria: χ^2^/*df* < 3 [45]; goodness-of-fit index > 0.80 [46]; adjusted goodness-of-fit index > 0.80 [47]; root mean square error of approximation < 0.08 [48]; Tucker–Lewis index > 0.90 [47]; and comparative fit index > 0.90 [48]. The results indicated a good model fit: χ^2^(520)/*df* = 1.864; goodness-of-fit index = 0.869; adjusted goodness-of-fit index = 0.849; root mean square error of approximation = 0.041; Tucker–Lewis index = 0.926; and comparative fit index = 0.932. Figure 2 illustrates the hypothesized serial mediation pathway, depicting the sequential influence of ICT use on self-management through health literacy, social support, and self-efficacy.

The serial mediation model revealed significant indirect effects of ICT use on self-management through the three mediators (*b* = 1.6094, 95% CI = 1.1188, 2.1314). Regarding direct effects, ICT use exhibited positive and significant associations with both health literacy (*b* = 1.4484, *p* < 0.001) and social support (*b* = 0.6857, *p* < 0.001); however, the direct relationship with self-efficacy was not statistically significant (*b* = 0.5225, *p* = 0.529). As presented in Table 3, health literacy had significant positive effects on social support (*b* = 0.3555, *p* < 0.001), self-efficacy (*b* = 0.3794, *p* < 0.001), and self-management (*b* = 0.5898, *p* < 0.001). Social support had positive impacts on self-efficacy (*b* = 0.2840, *p* < 0.001) and self-management (*b* = 0.2186, *p* < 0.001). Finally, self-efficacy had a conspicuous positive impact on self-management (*b* = 0.3490, *p* < 0.001). Thus, according to the joint significance test, the chain mediation effects from ICT use to self-management were significant.

As shown in Table 4, the indirect impact of the path containing ICT use, health literacy, social support, self-efficacy, and self-management was significant, and the 95% bootstrap CI did not include zero (*b* = 0.0510, *SE* = 0.0170, 95% CI = 0.0236, 0.0904). The total indirect effect was also significant (*b* = 1.6099, *SE* = 0.2598, 95% CI = 1.1188, 2.1314). the results are in accordance with the hypothesis that the positive impact of ICT use on self-management would be mediated by a series of mediating effects, as shown in Table 4. ICT use positively influenced health literacy (*b* = 1.4484, *p* < 0.001), which positively affected social support (*b* = 0.3555, *p* < 0.001), which positively influenced self-efficacy (*b* = 0.2840, *p* < 0.001), which, finally, influenced self-management (*b* = 0.3490, *p* < 0.001).

Table 5 presents 21 pairwise comparisons of the magnitudes of indirect effects. The magnitude of the indirect effect of ICT use on self-management mediated by health literacy was greater than that for six other indirect effect pathways. However, the magnitudes of the indirect effects mediated by social support, self-efficacy, and the remaining five indirect pathways did not significantly differ. The magnitude of the indirect effect via both health literacy and social support was larger than that via social support and self-efficacy.

## 4. Discussion

This study investigates the roles of health literacy, social support, and self-efficacy as mediators in the relationship between ICT use and self-management behavior among older adults with comorbidities. It further explores the specific functions of these variables in the mediating process. Based on the bootstrapping methodology recommended by Hayes [44], the findings demonstrate that health literacy, social support, and self-efficacy, as elicited by ICT use, serve as significant mediating factors influencing self-management behaviors among older adults with comorbidities.

Several key findings emerged from the results. Initially, the three hypothesized mediators (health literacy, social support, and self-efficacy) collectively mediated the relationship between ICT use and self-management, accounting for a substantial proportion (0.5473) of the overall indirect effect relative to the total effect. Consistent with prior research by Lee et al. [49] and Kong et al. [33], the findings indicate health literacy, social support, and self-efficacy function were statistically significant mediators influencing the development of self-management behavior among older adults with comorbidities. Furthermore, the serial mediation model analysis revealed that health literacy served as an initial and critical step in fostering older adults’ social support and highlighted its vital role in enhancing their self-management behavior. Aligning with the conclusions of Liu et al.’s [50] and Dinh and Bonner’s [20] research, this study suggests that older adults with comorbidities who acquire improved health literacy through health-related ICT use are more inclined to seek and engage in social support. This engagement, in turn, may facilitate self-efficacy by providing increased opportunities to express feelings and concerns and obtain knowledge regarding their conditions, thereby fostering greater confidence in managing their illness and leading to improved self-management behavior. In summary, self-efficacy encourages focused engagement in activities [51], which the literature has revealed to be an important attribute for older adults’ self-management development [52].

Interestingly, the results show that the magnitude of the indirect effect of ICT use on self-management via social support and self-efficacy was smaller than that via health literacy and self-efficacy. One potential explanation for this observation pertains to the complex nature of social support. Although social support has been identified as a significant factor in self-management development, research has also suggested that social support may have detrimental effects. Specifically, excessive social support may inadvertently foster a sense of dependence in patients, potentially weakening their perceived responsibility for self-management behaviors related to diet, medication adherence, and emotional regulation [52,53]. Another plausible explanation lies in the potential for suboptimal quality of social support, particularly if provided by individuals with limited health literacy. Individuals providing such low-quality support may not provide effective guidance and could even disseminate inaccurate information, potentially leading to adverse outcomes regarding self-management.

Another observation arising from the parallel mediation model is the significant contribution of self-efficacy to the overall indirect effect. However, in the serial mediation model, the specific indirect effect of ICT use on self-management solely through self-efficacy was not significant. This discrepancy may be attributed to the nature of serial modeling, wherein the influence of self-efficacy on self-management may have been partially suppressed or mediated by the preceding mediators (health literacy and social support). This suggests that self-efficacy does not emerge independently but is likely stimulated and consolidated through a sequential process involving enhanced health literacy and access to social support networks.

This issue is particularly relevant when examining the characteristics of ICT implemen-tation across various contexts. In high-income nations, systematic, integrated digital health programs can offer consistent reinforcement to patients with comorbidities. In numerous low- and middle-income countries, including China, ICT solutions tend to be more fragmented. Such fragmentation can create inconsistencies in digital health en-gagement and data flow. This could potentially impact the continuous and reliable sup-port essential for developing and sustaining an individual’s self-efficacy in self-management. Consequently, our findings emphasize the importance of utilizing these mediators to effectively enhance self-management among older adults with comorbidities in contexts where broader digital health integration is still in development.

### 4.1. Implications and Limitations

This study provides several research-related and practical insights and implications in the realm of ICT in healthcare. Theoretically, this study’s serial mediation model contributes to the literature by elucidating the pathways through which ICT use influences self-management, with health literacy acting as a critical initial mediator that fosters social support and self-efficacy. This model provides a valuable framework with which researchers can further explore the mechanisms and outcomes associated with health ICT use.

Methodologically, this study’s strengths include its development of an evidence-based mediation model, its focus on the understudied population of older adults with comorbidities (addressing a gap in single disease-focused research), and its rigorous statistical analysis using the PROCESS macro with bootstrapping, which offers enhanced power compared to traditional methods [54,55]. Although structural equation modeling is increasingly used in mediation analyses, offering the advantage of accounting for random measurement errors (a limitation of the observed-variable modeling method inherent in the PROCESS macro [56]), research has indicated that results are substantially similar when applying structural equation modeling and PROCESS to sufficiently large samples [56].

Practically, the findings of this study provide valuable insights into the relationship between ICT use and chronic disease management among older adults with comorbidities, especially in other deeply aging countries with similar Chinese cultural backgrounds. The empirical evidence suggests that among older adults with comorbidities, health literacy, social support, and self-efficacy, when facilitated by health-related ICT, contribute to a greater understanding of the development of self-management behavior in this population. This underscores the urgent need for medical practitioners, policymakers, and ICT designers to prioritize and implement such ICT-based interventions, a need further amplified by the COVID-19 pandemic’s shift toward remote chronic disease management [57]. In this context, increased reliance on ICT offers a valuable avenue for delivering high-quality and accessible content, such as health-related WeChat official accounts or TikTok video channels on smartphones or tablets, providing clear information, resources, and healthcare services. Furthermore, ICT can function as a cognitive tool, empowering older adults with comorbidities in active learning environments [58]. A key practical recommendation is for health educators to collaborate with designers to create clear and practical ICT content, particularly for individuals managing multiple conditions, potentially requiring a shift toward symptom- and function-focused self-management goals. Additionally, existing services, such as health and wellness, daily living, and support services, should be evaluated for their potential to undergo digital adaption. This would enhance accessibility and provide older adults with increased support for symptom management and health behavior maintenance. Concurrently, the development of programs like digital health literacy training and accessible community technology support is essential to decrease the impact of the digital divide on order adults.

Despite these notable strengths, caution is warranted when interpreting this study’s findings due to its cross-sectional design, so all the relationships found should be interpreted as associations at a single point in time. This fact inherently limits the establishment of temporal precedence among the variables in the proposed mediation model. We cannot definitely determine if ICT use leads to improved self-management or if individuals who are already better at self-management are simply more likely to use ICT. Future researchers employing longitudinal designs are recommended to rigorously test the temporal sequence and directionality of the mediational relationships identified. Another limitation is that our decision to recruit participants from a single hospital in Shanghai restricts the generalizability of the findings. This localized sampling approach may limit the applicability of the conclusions to rural or less-developed regions of China or even older populations in countries with different cultures and institutions. Therefore, it is suggested that future researchers should attempt to collect data across multiple institutions in diverse regions within a city, other provinces, or potentially international settings. Additionally, this study did not dive into the mechanisms by which service characteristics influence the interaction among the proposed mediators. Future research should investigate the characteristics of existing services and their impact on this pathway.

### 4.2. Conclusions

This research pinpoints factors associated with self-management among older adults with comorbidities in China and demonstrates a complex serial multiple-mediation path through health literacy, social support, and self-efficacy, influencing the relationship between health-related ICT use and self-management. This finding provides a valuable reference for developing targeted intervention programs aimed at enhancing self-management in this population. Specifically, these results provide a novel perspective and theoretical foundation for fostering internal cognitive and behavioral skills as well as leveraging external resources to increase inner confidence, which, in turn, can promote improved self-management adherence and contribute to sustained quality of life among older adults living with multiple chronic conditions.

## Figures and Tables

**Table 1:** Demographic characteristics of the study participants (*n* = 520).

Variable	*n* or *M*	% or *SD*
Age (range: 60–93)	70.80	6.30
Gender		
Male	262	50.4
Female	258	49.6
Marital status		
Married	414	79.6
Never married, divorced, bereaved	106	20.4
Income		
>2590	492	94.6
≤2590	28	5.6
Education		
<High school	218	41.9
High school	198	38.1
>High school	104	20.0
Living status		
Alone	57	11.0
With others	463	89.0
Activities of daily living	80.77	15.32
Number of chronic diseases	3.71	1.54
Depression symptom	3.31	3.84
Self-rated health (range: 1–5)	3.17	0.83
ICT use	−0.01	1.44
Health literacy	48.63	6.95
Social support	23.88	7.55
Self-efficacy	39.02	12.32
Self-management (range: 0–88)	68.14	12.81

**Table 2:** Correlations between ICT use, health literacy, social support, self-efficacy, and self-management.

Variable	ICT Use	Health Literacy	Social Support	Self-Efficacy
Health literacy	0.385 *			
Social support	0.279 *	0.413 *		
Self-efficacy	0.223 *	0.419 *	0.354 *	
Self-management	0.363 *	0.527 *	0.418 *	0.514 *

* *p* < 0.001.

**Table 3:** Mediation effects (bootstrapping 5000 samples).

Path	*b*	95% CI
ICT use → health literacy → self-management	0.8543 *	0.5399, 1.2097
ICT use → social support → self-management	0.2624 *	0.0978, 0.4808
ICT use → self-efficacy → self-management	0.4932 *	0.1926, 0.8135

* *p* < 0.001.

**Table 4:** Total direct, direct, total indirect, and indirect effects and pairwise comparisons between indirect effects with 95% bootstrap confidence intervals.

Path ^1^	*b*	*SE*	95% CI
Total effect	2.9413	0.3851	2.1848, 3.6978
Direct effect	1.3314	0.3404	0.6629, 2.0002
Indirect effects	1.6099	0.2598	1.1188, 2.1314
Ind1: ICT → HL → SM	0.8543	0.1681	0.5422, 1.2022
Ind2: ICT → SS → SM	0.1499	0.0734	0.0312, 0.3142
Ind3: ICT → SE → SM	0.1824	0.1420	−0.1002, 0.4636
Ind4: ICT → HL → SS → SM	0.1126	0.0380	0.0465, 0.1955
Ind5: ICT → HL → SE → SM	0.1918	0.0599	0.0879, 0.3252
Ind6: ICT → SS → SE → SM	0.0680	0.0314	0.0155, 0.1378
Ind7: ICT → HL → SS → SE → SM	0.0510	0.0170	0.0236, 0.0904

^1^ ICT = information and communication technology. HL = health literacy. SE = self-efficacy. SM = self-management. SS = social support.

**Table 5:** Pairwise comparisons between indirect effects with 95% bootstrap confidence intervals.

Pairwise Comparisons of Indirect Effects	*b*	*SE*	95% CI
Ind1–Ind2	0.7044	0.1918	0.3348, 1.0861
Ind1–Ind3	0.6719	0.2202	0.2474, 1.1061
Ind1–Ind4	0.7418	0.1689	0.4246, 1.0874
Ind1–Ind5	0.6625	0.1593	0.3687, 0.9923
Ind1–Ind6	0.7863	0.1716	0.4678, 1.1413
Ind1–Ind7	0.8033	0.1643	0.4987, 1.1470
Ind2–Ind3	−0.0325	0.1640	−0.3587, 0.2989
Ind2–Ind4	0.0373	0.0686	−0.0930, 0.1796
Ind2–Ind5	−0.0419	0.0975	−0.2243, 0.1538
Ind2–Ind6	0.0819	0.0628	−0.0156, 0.2259
Ind2–Ind7	0.0988	0.0793	−0.0354, 0.2721
Ind3–Ind4	0.0698	0.1475	−0.2215, 0.3630
Ind3–Ind5	−0.0094	0.1588	−0.3400, 0.3001
Ind3–Ind6	0.1144	0.1457	−0.1765, 0.4007
Ind3–Ind7	0.1313	0.1416	−0.1497, 0.4100
Ind4–Ind5	−0.0792	0.0698	−0.2245, 0.0485
Ind4–Ind6	0.0446	0.0540	−0.0612, 0.1496
Ind4–Ind7	0.0615	0.0398	−0.0129, 0.1450
Ind5–Ind6	0.1238	0.0659	0.0060, 0.2641
Ind5–Ind7	0.1408	0.0589	0.0364, 0.2685
Ind6–Ind7	0.0169	0.0302	−0.0415, 0.0788

## Data Availability

The data is unavailable due to ethical restrictions.

## Abbreviations

The following abbreviations are used in this manuscript:

ICT	Information and communication technology
